# Validación del puntaje de valoración de la gravedad de la enfermedad pulmonar obstructiva crónica en población colombiana en un servicio de atención primaria

**DOI:** 10.7705/biomedica.5123

**Published:** 2020-12-10

**Authors:** Jorge Mario Estrada-Álvarez, Juan Pablo Orozco-Hernández, Luis Evelio Aristizábal-Franco

**Affiliations:** 1 Grupo de Investigación en Salud Comfamiliar, Clínica Comfamiliar, Pereira, Colombia Grupo de Investigación en Salud Comfamiliar Clínica Comfamiliar Pereira Colombia; 2 Programa de Medicina, Universidad Tecnológica de Pereira, Pereira, Colombia Universidad Tecnológica de Pereira Programa de Medicina Universidad Tecnológica de Pereira Pereira Colombia; 3 Grupo de Investigación en Gerencia del Cuidado, Universidad Libre de Pereira, Pereira, Colombia Grupo de Investigación en Gerencia del Cuidado Universidad Libre de Pereira Pereira Colombia

**Keywords:** enfermedad pulmonar obstructiva crónica, índice de gravedad de la enfermedad, atención primaria de salud, espirometría, encuestas y cuestionarios, Colombia, Pulmonary disease, chronic obstructive, severity of illness index, primary health care, spirometry, surveys and questionnaires, Colombia

## Abstract

**Introducción.:**

La enfermedad pulmonar obstructiva crónica (EPOC) es un importante problema de salud mundial con una alta morbimortalidad. Se requiere la medición de la gravedad de la enfermedad mediante una herramienta de fácil aplicación, bajo costo y fácil disponibilidad en áreas rurales.

**Objetivo.:**

Evaluar la validez y contabilidad del puntaje de valoración de la gravedad de la EPOC *(Chronic Obstructive Pulmonary Disease Severity Score,* COPDSS) en una población de atención primaria en Colombia.

**Materiales y métodos.:**

Se hizo un estudio de corte transversal en una muestra de 100 pacientes con diagnóstico de EPOC según las guías GOLD. La validez concurrente se evaluó correlacionando los resultados del COPDSS con otras variables de importancia como las del cuestionario sobre la enfermedad respiratoria crónica *(Chronic Respiratory Disease Questionnaire,* CRQ) y el volumen espiratorio forzado en el primer segundo *(Forced Expiratory Volume in one second,* FEV_1_).

**Resultados.:**

El COPDSS presentó una correlación significativa con el FEV_1_ (r=-0,33), con el CRQ (r=-0,57) y sus dimensiones: disnea (r=0,51), fatiga (r=0,53), función emocional (r=0,43) y control de la enfermedad (r=0,50). En el análisis factorial se determinó un solo factor con una varianza acumulada de 59,1 %. El análisis de coherencia interna mostró un alfa de Cronbach de 0,76, valor este considerado adecuado.

**Conclusiones.:**

Se observó que el uso del COPDSS como cuestionario de valoración de la gravedad de pacientes con EPOC en Colombia tenía validez y confiabilidad adecuadas y que es de fácil aplicación en la atención primaria.

## Abstract (en)

**Introduction:**

Chronic obstructive pulmonary disease (COPD) is a major global health problem causing high morbidity and mortality. Easy to apply, cost-effective, and available COPD severity measures are required in rural areas.

**Objective:**

To assess the validity and reliability of the COPD Severity Score (COPDSS) in Colombian patients attended in primary care.

**Materials and methods:**

A cross-sectional study was conducted in a sample of 100 patients diagnosed with COPD according to GOLD guidelines. Convergent validity was assessed by correlating the COPDSS results with other important variables such as those assessed through the Chronic Respiratory Disease Questionnaire (CRQ) and the forced expiratory volume in one second (FEV_1_).

**Results:**

The COPDSS showed a significant correlation with the FEV_1_ (r=-0.33), with the CRQ (r=-0.57) and its dimensions: Dyspnea (r=0.51), fatigue (r=0.53), emotional function (r=0.43), and disease control (r=0.50). The factor analysis identified a single factor with a cumulative variance of 59.1%. The internal consistency analysis showed an adequate Cronbach's alpha of 0.76.

**Conclusions:**

The use of the COPDSS as an assessment test for COPD patients in Colombia showed adequate validity, reliability, and easy application in primary care.

La enfermedad pulmonar obstructiva crónica (EPOC) es un importante problema de salud mundial con una alta morbimortalidad ^1^. Actualmente, la EPOC es la tercera causa de muerte en el mundo, ocupa el quinto lugar en términos de carga de la enfermedad, tiene un riesgo acumulado de por vida estimado del 25 %, y afecta a hombres y mujeres por igual ^2^. En Colombia, se estimó una prevalencia del 8,9 % en cinco ciudades para el 2007 ^3^ y una importante carga de la enfermedad, con 208.166 años perdidos de vida ajustados por discapacidad para el 2015, ocupando el tercer lugar en Latinoamérica después de México y Argentina ^1^.

En general, las tasas de mortalidad por EPOC específicas para la edad están disminuyendo, y el aumento global en el número de muertes se relaciona con el crecimiento y el envejecimiento de la población, ya que la enfermedad afecta predominantemente a los ancianos ^1^. En los países de altos ingresos, el tabaquismo es el principal factor de riesgo para el desarrollo de la EPOC, pero también se reconocen otros factores entre los que se destacan la exposición al humo de la biomasa, las infecciones en la niñez, los factores genéticos y el asma alérgica, especialmente en los países de ingresos bajos y medios como Colombia, Argentina, China y Brasil, entre otros ^4^. Aunque se han logrado avances en el tratamiento de la EPOC, es necesario encontrar terapias que reduzcan la progresión de la enfermedad y la mortalidad ^5^.

Actualmente, hay diversos métodos para clasificar la gravedad de los pacientes con EPOC con fines de pronóstico y tratamiento, recurriendo, entre otros aspectos, a la valoración de la función pulmonar alterada, la disnea grave, el tabaquismo activo y el puntaje de gravedad de la EPOC *(Chronic Obstructive Pulmonary Disease Severity Score,* COPDSS) ^6^.

El COPDSS es un cuestionario corto y de fácil aplicación desarrollado por Eisner, *et al.*^6^, ya traducido y validado en español ^7^. El COPDSS ha demostrado tener una buena validez y capacidad predictiva para exacerbaciones por la EPOC, con mejores resultados predictivos que otras variables ^8^. La clasificación de la gravedad y del riesgo de los pacientes con un método fácil y práctico es de relevancia para la práctica clínica, ya que la disponibilidad de la espirometría puede ser limitada en áreas de difícil acceso o en zonas rurales. Asimismo, la implementación de un método de bajo costo y mayor accesibilidad permitiría un mejor manejo de la EPOC, con intervenciones más tempranas y pertinentes, reduciendo la morbilidad, la mortalidad y los costos asociados con la atención en salud de estos pacientes a largo plazo.

En Colombia, las guías de práctica clínica no incluyen métodos de clasificación de la gravedad de fácil y rápida implementación, y el COPDSS no ha sido validado en el país. Asimismo, en los países de bajos y medianos ingresos, la gravedad y los factores de riesgo asociados con la EPOC pueden ser diferentes a los de los países de mayores ingresos.

Dada la necesidad de un método de clasificación de la gravedad de la EPOC de fácil aplicación y acceso, el objetivo de este estudio fue validar el COPDSS en una población de Colombia con diagnóstico de EPOC confirmado por espirometría.

## Materiales y métodos

### Pacientes y muestra

Se desarrolló un estudio de corte transversal con enfoque de validación de instrumentos basado en lo sugerido por diferentes autores ^9^^-^^11^. Se seleccionaron 100 pacientes pertenecientes a un programa de EPOC, todos con confirmación del diagnóstico mediante espirometría según las guías GOLD ^12^.

Los criterios de inclusión fueron: una edad igual o mayor de 40 años; diagnóstico de EPOC según los criterios de la guía GOLD; índice FEV_1_/ FVC<0,7 en la espirometría posterior a la broncodilatación, y presencia de alguno de los siguientes factores de riesgo: exposición a humo de tabaco, de leña, o exposición ocupacional respiratoria a partículas, además de síntomas respiratorios crónicos (disnea, tos o expectoración).

Se excluyeron aquellos pacientes con algún déficit cognitivo que impidiera la respuesta adecuada del cuestionario y aquellos con una espirometría que no cumplía con los criterios de buena calidad.

El tamaño de la muestra se definió siguiendo la recomendación de Streiner, *et al.*^11^^),^ es decir, elegir entre 5 y 7 individuos por ítem del cuestionario y garantizar una muestra no menor de 100 individuos.

El estudio fue aprobado por el Comité de Bioética del Hospital de Santa Mónica (Dosquebradas, Risaralda) como un estudio "sin riesgo" según la Resolución 8430 de 1993 expedida por el Ministerio de Salud de Colombia y acorde con la Declaración de Helsinki. Todos los pacientes firmaron el consentimiento informado aceptando participar en la investigación.

### Recolección de información

Siguiendo los procedimientos de validación propuestos para este tipo de cuestionarios, se hizo la traducción al español. Aunque ya se había validado este cuestionario en España ^7^, debido a las diferencias lingüísticas con el español de Colombia y a las de algunos medicamentos empleados en el país, fue necesaria una traducción ajustada al contexto nacional ^11^. En primer lugar, la versión original del cuestionario fue traducida por un profesional bilingüe experto en el área específica, versión que fue revisada después por otro profesional bilingüe para reducir el riesgo de errores de traducción.

El cuestionario consta de cinco aspectos generales por evaluar: síntomas respiratorios, con un máximo de 7 puntos; uso de corticoides, con un máximo de 5 puntos; uso de otros medicamentos, con un máximo de 10 puntos, y hospitalización o uso de oxígeno domiciliario, con un máximo de 13 puntos, para un puntaje total en un rango entre 0 y 35 puntos en el que los puntajes más altos se asocian con una mayor gravedad de la EPOC. Cada ítem tiene un peso *a priori* basado en aspectos clínicos de la enfermedad y su contribución esperada en la gravedad general de la EPOC ^6^.

Cada paciente fue citado a una consulta clínica para valoración que incluyó una entrevista cara a cara utilizando el cuestionario y preguntas sobre los datos sociodemográficos y aspectos clínicos de la enfermedad. La información fue recolectada por profesionales en terapia respiratoria entrenadas previamente en la aplicación de los cuestionarios y en la toma de espirometrías. La espirometría se ajustó a las guías de la *American Thoracic Society* (ATS) y la *European Respiratory Society* (ERS) ^13^.

### Validación

Como parte del proceso de validación concurrente, se plantearon diferentes hipótesis de relación que permitieran dar cuenta de la variable latente de gravedad de la enfermedad, para lo cual se consideraron como parámetros clínicos la espirometría y la calidad de vida relacionada con la salud. Esta última se midió mediante el *Chronic Respiratory Questionnaire* (CRQ) desarrollado por Guyatt, *et al.*^14^, y validado en Colombia previamente ^15^. Este cuestionario también fue administrado por profesionales en terapia respiratoria previamente entrenadas en su diligenciamiento.

El CRQ se compone de 20 preguntas o ítems y se divide en cuatro dimensiones de la calidad de vida relacionada con la salud: disnea, fatiga, función emocional y control de la enfermedad. El paciente selecciona una respuesta para cada ítem a partir de una escala de siete posibles respuestas equidistantes. Se puntúan por separado cada una de las dimensiones y también se obtiene una puntuación total. En el CRQ las puntuaciones más altas indican una mejor calidad de vida.

### Análisis estadístico

Se hizo un análisis estadístico descriptivo con medidas de tendencia central expresadas como medias y medianas, y las medidas de dispersión, como varianza, desviación estándar y percentiles. Para la evaluación de la validez del constructo, se hizo un análisis factorial exploratorio con extracción por el método de componentes principales usando como criterio la selección de factores con valores propios mayores de 1; la rotación de los factores extraídos se hizo mediante el método varimax con normalización de Kaiser.

En todos los análisis se cumplieron previamente los supuestos para la aplicación del método, es decir, una prueba de esfericidad de Barttlet significativa y la medida de adecuación de Kaiser-Meyer-Olkin.

La correlación bivariada entre el puntaje del cuestionario de gravedad y las otras medidas clínicas, se hizo mediante los coeficientes de correlación de Spearman y de correlación parcial. La coherencia interna (confiabilidad) se estimó con el alfa de Cronbach. En todos los análisis se utilizó un nivel de significación de 0,05. Se utilizó el programa Stata 14.0™ para los análisis estadísticos.

## Resultados

### Características clínicas de los pacientes

Se incluyeron 106 pacientes para la validación del COPDSS, de los cuales se excluyeron seis debido a espirometrías que no cumplían con los criterios de calidad, es decir, el análisis se hizo en 100 pacientes.

La edad promedio fue de 73,4 ± 9,2 años y el 52 % pertenecía al sexo femenino. La distribución por estado civil fue la siguiente: solteros, 32 %, casados, 23 % y viudos, 24 %.

Los parámetros espirométricos mostraron un promedio de porcentaje predicho en el VEF_1_ (volumen espiratorio forzado al primer segundo) de 59,3 % y de 68,4 % en la capacidad vital forzada.

En cuanto al CRQ, los valores cercanos a siete evidenciaban la mejor calidad de vida y, aquellos cercanos a uno, la peor. En todas las dimensiones se obtuvo un puntaje promedio por encima de cinco, lo que indicaba una buena calidad de vida entre los evaluados. Otras características clínicas de los pacientes se pueden ver en el cuadro 1.

**Cuadro 1:** Principales características clínicas de los pacientes con enfermedad pulmonar obstructiva crónica (EPOC) (n=100)


Edad (años) (media ± DE)	73,4 ± 9,2
Sexo (%)	
Masculino	48
Femenino	52
Terapia por inhalación (%)	
Agonistas beta-2 de acción corta	42
Agonistas beta-2 de acción prolongada	4
Anticolinérgico	15
Corticoesteroides	12
Oxigeno domiciliario (%)	12
Horas/día oxígeno, media ± (DE)	10 ± 7
Tabaquismo (paquete/año), media ± (DE)	40 ± 125
VEF_1_, media ± (DE)	59,3 ± 27,1
CRQ, media ± (DE)	
Disnea	6 ± 1
Fatiga	5 ± 1
Función emocional	5 ± 2
Control de la enfermedad	5 ± 2

DE: desviación estándar; VEF_1_: Volumen espiratorio forzado al primer segundo; CRQ: *Chronic Respiratory Questionnaire*

En cuanto a los resultados del COPDS, el puntaje objetivo mostró una distribución asimétrica (prueba de Kolmorogov, p<0,0001), con una mediana de 7 (rango intercuartílico, RIC=4-12). La proporción de respuestas para cada ítem evaluado se presenta en el cuadro 2.

**Cuadro 2:** Ítems en el cuestionario de gravedad de la enfermedad pulmonar obstructiva crónica (EPOC)

Item	Puntaje	%
Síntomas respiratorios (máximo, 7 puntos)		
Ninguno	0	21
Caminando de prisa o apurado en terreno plano	1	25
Caminando con otra persona de su misma edad en terreno plano	2	0
Tiene que detenerse para tomar aire cuando camina a su ritmo en un nivel plano	3	54
Disnea en los pasados 14 días o noches		
Ninguna	0	34
1 a 2 días o noches	1	14
3 a 6 días o noches	2	26
7 a 13 días o noches	3	15
Todos los días o noches	4	11
Uso de corticoide sistémico (máximo, 5 puntos)		
Alguna vez lo ha usado	1	18
Usado en el pasado año	3	17
Usado en las pasadas dos semanas	1	20
Uso de otra medicación (máximo, 10 puntos) Inhaladores en las pasadas dos semanas		
Agonistas beta 2 de corta acción	1	38
Agonistas beta 2 de acción prolongada	1	7
Corticoide inhalado	1	16
Anticolinérgicos	1	25
Uso de nebulizador en las pasadas dos semanas		
Agonista beta 2 de acción corta	1	6
Anticolinérgicos	1	9
Medicación oral		
Teofilina en las pasadas dos semanas	1	5
Agonistas beta 2	1	1
Antibiótico para condiciones pulmonares en los pasados 12 meses		
Uno a dos periodos	1	13
Tres o más periodos	2	87
Hospitalización, intubación, uso de oxígeno domiciliario (máximo, 13 puntos)		
Hospitalizado por EPOC en los pasados cinco años	3	39
Intubado por EPOC en los pasados cinco años	5	1
Oxigeno domiciliario actualmente	5	11

### Validez del constructo

En la evaluación de esta forma de validez, el análisis factorial dio como resultado una matriz de correlaciones significativas según la prueba de Bartlett (ji al cuadrado=126,53; p=0,0001), con un coeficiente de adecuación de de Kaiser-Meyer-Olkin de 0,7, lo que indicó que el método de análisis factorial podía aplicarse a los datos del estudio.

En la determinación de la estructura interna de la escala, que permite el establecimiento de la variable latente (en este caso, la gravedad de la enfermedad), se encontró un solo factor con valor propio de 2,36 y una proporción de varianza acumulada explicada por este factor de 59,1 %. Las cargas factoriales, que indican el peso que tiene cada área sobre el factor (gravedad de la enfermedad) de cada una de las áreas de evaluación en el cuestionario, se muestran en el cuadro 3.

**Cuadro 3:** Cargas factoriales de cada área de evaluación de la escala de gravedad de la enfermedad pulmonar obstructiva crónica (EPOC)

Áreas	Factor 1
Síntomas respiratorios	0,59
Uso de medicamentos	0,89
Hospitalización, intubación, oxígeno	0,84
Uso corticoide sistémico	0,69

### Validez concurrente: correlación con otros parámetros

En el análisis de asociación con parámetros de la función pulmonar, el VEF_1_ tuvo una correlación significativa con el puntaje de gravedad del cuestionario (r=-0,33; p=0,0008) después de controlar por la edad. En el cuadro 4 se presentan las correlaciones entre la calidad de vida relacionada con la salud y las dimensiones de la misma según el COPDSS, controlando por los valores del porcentaje predicho del VEF_1_ y la edad. Se presentaron correlaciones moderadas con las dimensiones de disnea y de fatiga, y débiles con las de función emocional y control de la enfermedad.

**Cuadro 4:** Correlación entre los puntajes de la calidad de vida relacionada con la salud (CRQ) y escala de gravedad de la enfermedad (COPDSS)

Calidad de vida relacionada con la salud	Coeficiente correlación no ajustado	Coeficiente de correlación ajustado^*^
Puntaje total en el CRQ	-0,48	-0,57
Disnea	-0,49	-0,51
Fatiga	-0,50	-0,53
Función emocional	-0,37	-0,46
Control de la enfermedad	-0,38	-0,50

^*^Valor del coeficiente de correlación parcial ajustado por VEF_1_ y edad. Todos los análisis fueron estadísticamente significativos (p<0,001).

### Contabilidad (coherencia interna)

La correlación entre los ítems de la escala, la cual muestra la coherencia interna del cuestionario, dio como resultado un alfa de Cronbach de 0,76, que corresponde a una confiabilidad adecuada.

## Discusión

La EPOC es una de las principales causas de mortalidad y morbilidad a nivel mundial, con una alta carga de la enfermedad y costos para el sistema de salud ^1^. En Colombia, se han hecho algunos estudios en esta población ^3^^,^^15^^-^^19^, pero hasta el momento no se había validado una escala de gravedad de la EPOC, siendo esta la primera validación en el país. Este estudio en una población de pacientes con EPOC confirmada por espirometría demuestra que el uso del COPDSS es factible, de fácil aplicación en los servicios de atención primaria, con una adecuada correlación con la calidad de vida y la función pulmonar, y con una coherencia interna apropiada.

Actualmente, la estratificación de la gravedad de la EPOC se hace con base en parámetros fisiológicos, como la función pulmonar (FEV_1_) según las guías GOLD ^12^, y en los síntomas de disnea y de exacerbación ^20^ que, aunque son medidas de gravedad de la enfermedad, no tienen en cuenta resultados de relevancia como la calidad de vida relacionada con la salud y la discapacidad. De ahí la relevancia de la adopción de nuevas formas de medición de la gravedad de la enfermedad con base en múltiples dimensiones y aspectos, como el uso de inhaladores, la necesidad de oxígeno domiciliario, las exacerbaciones que conlleven la hospitalización en unidades de cuidados intensivos y la reducción de síntomas.

Asimismo, otras medidas utilizadas en estudios clínicos, como la caminata de seis minutos ^21^ y el índice BODE ^22^, entre otros ^20^, deben aplicarse en un ámbito clínico, lo cual limita su uso en el marco de encuestas poblacionales epidemiológicas. A esto se suma que la determinación de la gravedad mediante la espirometría puede ser costosa y no estar disponible en zonas de difícil acceso o en áreas rurales. Se ha establecido que, a diferencia de quienes viven en zonas urbanas, en pacientes con EPOC de zonas rurales la mortalidad y la prevalencia de EPOC son mayores ^23^^,^^24^, y que las fallas en el diagnóstico conllevan un tratamiento menos efectivo ^25^.

En este estudio, el COPDSS demostró tener validez concurrente, pues hubo correlaciones significativas con parámetros como la calidad de vida y la función pulmonar (VEF_1_) de igual magnitud y dirección a las reportadas en el estudio inicial de validación y en otros posteriores ^6^. Además, según el análisis factorial exploratorio, se verificó la validez del constructo al determinar que en su estructura interna los ítems se constituyeron en un solo factor que explicaría el 59,7 % de la variabilidad total del fenómeno estudiado (gravedad de la enfermedad). También su coherencia interna demostró ser adecuada y similar a la previamente reportada ^6^^,^^7^.

Con base en estos resultados y los de estudios previos ^6^^,^^7^, el COPDSS puede utilizarse como una herramienta de clasificación de la gravedad de la EPOC muy valiosa, principalmente en zonas rurales o de difícil acceso, pues permite una fácil y rápida clasificación del paciente en los servicios de atención primaria. Este método no remplaza la espirometría, aunque es una útil herramienta en zonas donde no se dispone de esta prueba. Además, en España, por ejemplo, se evidenció que en la práctica clínica no se utilizaba regularmente la espirometría hasta en el 41 % de los encuestados ^7^.

Por otra parte, el COPSS puede emplearse conjuntamente con otros cuestionarios para ayudar en el diagnóstico y seguimiento de los pacientes con EPOC ^7^. Se recomienda utilizarlo cada seis meses como parte de los programas de manejo de la EPOC para evaluar la evolución de la enfermedad de una forma más integral, además, podría ser útil en la investigación clínica como indicador de otro resultado importante por analizar en esta población.

En cuanto a la población estudiada, el 52 % correspondía al sexo femenino, es decir que hubo una relación de 1:1 entre los sexos, similar a la del estudio de validación original desarrollado por Eisner, *et al.*^6^, en tanto que, en la validación previa en España ^7^, predominó el sexo masculino (92 %) con una relación de 1:10. La edad promedio de los pacientes de este estudio fue un poco mayor a la de los pacientes del estudio original y a los de la validación en España ^6^^,^^7^. A pesar de estas diferencias, y dado que la edad y el sexo no se han asociado con la gravedad de la EPOC, la validez del instrumento es adecuada.

Es importante destacar, como lo hacen diferentes autores ^9^^-^^11^, que la validación de un instrumento es un proceso que se nutre de diferentes perspectivas y no un componente de "todo o nada" En este sentido, esta es una primera versión de validación del cuestionario; específicamente en el contexto de la validez concurrente, es necesario aportar evidencias a partir de otras formas de validación que aporten al proceso y a la consolidación de una medida más confiable en nuestra población que evalúe la validez predictiva y la de constructo bajo la modalidad de grupos extremos que den fuerza a las inferencias basadas en los resultados del cuestionario.
